# Living Near to Attractive Nature? A Well-Being Indicator for Ranking Dutch, Danish, and German Functional Urban Areas

**DOI:** 10.1007/s11205-016-1375-5

**Published:** 2016-06-03

**Authors:** Michiel N. Daams, Paolo Veneri

**Affiliations:** 1 Department of Economic Geography, Faculty of Spatial Sciences, University of Groningen, P.O.B. 800, 9700 AV Groningen, The Netherlands; 2 Regional Development Policy Division, OECD, 2 rue André-Pascal, 75775 Paris Cedex 16, France

**Keywords:** Comparative indicators, Functional urban areas, Natural amenities, Well-being, Population-weighted

## Abstract

While nature is widely acknowledged to contribute to people’s well-being, nature based well-being indicators at city-level appear to be underprovided. This study aims at filling this gap by introducing a novel indicator based on the proximity of city-residents to nature that is of high-amenity. High-amenity nature is operationalized by combining unique systematic data on people’s perceptions of what are the locations of attractive natural areas with data on natural land cover. The proposed indicator departs from the usual assumption of equal well-being from any nature, as it approximates the ‘actual’ subjective quality of nature near people’s homes in a spatially explicit way. Such indicator is used to rank 148 ‘cities’ in the Netherlands, Denmark, and Germany. International comparability of the indicator is enhanced by the use of a definition of cities as functional urban areas (FUAs), which are consistently identified across countries. Results demonstrate that the average ‘nearness’ of FUA populations to high amenity nature varies widely across the observed FUAs. A key finding, that complements insights from existing city-level indicators, is that while populations of FUAs with higher population densities may live relatively far from nature in general, they also live, on average, closer to high-amenity nature than inhabitants of lower density FUAs. Our results may stimulate policy-debates on how to combine urban agglomeration with access to natural amenities in order to account for people’s wellbeing.

**Electronic supplementary material:**

The online version of this article (doi:10.1007/s11205-016-1375-5) contains supplementary material, which is available to authorized users.

## Introduction

The attractiveness of distinct cities as places to live depends strongly on the presence of various amenities (Carlino and Saiz 2008; Florida et al. 2011; Glaeser et al. 2001). Residential location choices are in recent literature found to be increasingly driven by amenities (Chi and Marcouiller 2011). By the end of the century, the world’s population as a whole may be living in cities (see Batty 2011), and for this reason it will become ever more important to monitor the presence of local amenities that influence the well-being of city dwellers. The resulting information on the presence of local amenities supports empirical research on how well-being is shaped locally, and may inform decision-making with regard to local and regional development policies (European Commission 2014; Noll 2002; Waltert and Schläpfer 2010). To these ends, various institutions including the UN, Eurostat, and the OECD, maintain international databases of a wide range of city-level indicators of well-being. The scope of such well-being indicators varies from accessibility to hospitals to local crime rates. In the scientific literature there is also thoroughgoing discussion on how to measure and assess the live-ability, well-being or quality of life of urbanites who rely on different amenities (e.g. Hagerty et al. 2001; Liao 2009). This paper contributes to the assessment of urban well-being and natural amenities.

Natural amenities may add to urban inhabitants’ well-being significantly, as empirical studies show: natural amenities lead to higher prices of nearby houses, they attract interregional migration flows, and they increase the happiness and life satisfaction of individuals (Bertram and Rehdanz 2015; MacKerron and Mourato 2013; Waltert and Schläpfer 2010). However, while a growing field of literature studies how natural amenities relate to the well-being of urban inhabitants on a micro-level, indicators that aggregate measures from such studies to city-level, to allow comparison between cities, appear to be underprovided. Also, the OECD (2014, p. 69) notes a “current lack of internationally comparable indicators on environmental outcomes”. As such, policy makers may have limited reference for how green cities should be, and how concentrated or dispersed, while the relation between urban agglomeration and natural areas has also been subject to continuous and ongoing debate amongst academics ever since Howard wrote about the Garden City (e.g. Breheny 1996; Kühn 2003). This indicates a need for comparative city-level indicators that provide information on the presence of natural amenities that add to people’s well-being. We focus our work here on improving the measurement of what may be called the ‘level of appreciation of natural amenities in cities’.

The natural amenity indicators used in studies of urban well-being can be divided into the two main categories of subjective and objective. *Subjective* indicators approximate well-being through the degree to which urban inhabitants are satisfied or dissatisfied with natural amenities in their living environments (e.g. Noll 2002; Terzi et al. 2015); this type of indicator remains implicit about the physical environment to which the observed judgments refer, as it does not capture which precise natural places are judged. In contrast, *objective* indicators capture the physical presence of natural amenities within cities (e.g. green space per capita in Zanella et al. 2015, or the share of natural land use in a city in Lopes and Camanho 2013), but assume that all nature has an equal impact, if any, on the well-being of city inhabitants. Clearly, the information captured in existing objective and subjective indicators does not necessarily overlap, and may or may not signal the same message. To overcome gaps on either side of an indicator, *both* types have thus far been included as complements in frameworks of well-being indicators. Objective and subjective indicators have their own merits which are, respectively, spatial explicitness (how *close* are people to—any—nature?) and perceptual precision (do people *appreciate*—regardless of where precisely it is—nature that is nearby?). We strongly think that combining these merits into a single indicator will not only shed light on a critically policy-relevant issue, well-being in cities, but it will also increase our understanding of the attractiveness of cities as places to live: how *close* are people to nature of highly *appreciated* natural amenity? We therefore determine to answer this question by recognizing the importance of indicators that integrate both objective and subjective data (Marans 2015; OECD 2014).

The contribution of this paper to the literature on city-level well-being indicators is twofold. First, while existing nature based well-being indicators draw on either objective or subjective data, both these types of data are integrated in the well-being indicator that this paper introduces. Specifically, our indicator is set out to capture (objectively) *how near* the inhabitants of distinct cities are living to nature which may generate positive well-being outcomes: nature that is (subjectively) *perceived* to be of high-amenity. High-amenity nature is defined based on a systematic integration of natural land use data and data that capture the locations of nature that people find highly attractive. In doing so, we build on a measurement approach introduced in Daams et al. (2016). Their approach separates ‘high-amenity’ nature from lower amenity nature amongst natural land use data, country-wide, in a way that is empirically shown to be consistent with the perception of Dutch homebuyers. Important here is that the Daams et al. study demonstrates that high-amenity nature adds to house prices over a distance that is wider than has been indicated thus far in any house price study, due to such nature’s subjective quality. This strong relationship with house prices underpins our focus on high-amenity nature as a key aspect to urban well-being. In this work, data on the locations of high-amenity nature are combined with population data, which makes it possible to establish how near the inhabitants of distinct cities are living to high-amenity nature. High-amenity nature may include both large urban parks and water bodies as well as large natural areas situated outside city boundaries. As these typically cover wide swathes of land, high-amenity nature is not considered to be a substitute for urban nature of lower amenity such as small neighborhood parks with playgrounds or tree-lined streets. These types of nature may matter to people’s well-being on a local scale, whereas our indicator captures whether cities comprise nature, or are regionally embedded in nature that offers a high-quality amenity. This adds a complementary focus to the existing range of nature-based indicators (c.f. Lopes and Camanho 2013; Terzi et al. 2015; Zanella et al. 2015).

As a second contribution, we adopt a new definition of the borders of cities based on *functional* urban regions (see OECD 2012) in order to achieve maximum international comparability of the city-level indicator, which the present paper introduces. We use functional urban areas (FUAs) rather than the more arbitrary municipal boundaries at the core of most regional well-being indicators (e.g. Colombo et al. 2014), which may easily exclude nature at city fringes if the municipal boundaries are tightly defined. While tightness of municipal borders may indeed differ widely across cities, the definition of cities as *functional urban areas* ensures a consistent delineation of distinct urban areas’ borders. As a result, the outcomes of the high-amenity nature indicator constructed here can be compared consistently across FUAs, even when located in different countries. FUAs in the Netherlands, Denmark, and Germany are observed in our analysis because we have data for these countries from which we can define high-amenity nature.

Overall, this paper links the literature on internationally comparable well-being indicators with literature on the spatial assessment of environmental quality (Brown and Kyttä 2014) and the definition of urban areas (Brezzi and Veneri 2015). In doing so, this study makes manifest part of the research agenda outlined by James et al. (2009, pp. 70–71), which prioritizes research on “what are appropriate indicators and typologies for the comparative assessment, monitoring and prediction of the state and trends of urban green spaces.” In addition, the data generated from our study may support research on “what global competitive gains are delivered to cities through the provision of high-quality green spaces.” We thus propose that our research here serve as a building block in the wider literature on urban competitiveness and well-being from nature.

We structure the remainder of the paper as follows. The next section discusses theoretical considerations in the operationalization of natural amenity indicators. The study area and data are then discussed in Sect. 3, followed by a description of the methodology in Sect. 4. Results are presented and elaborated in Sect. 5, and Sect. 6 provides a concluding discussion.

## Building Natural Amenity Indicators

### Defining Natural Amenity-Levels

The operationalization of ‘high-amenity nature’ requires us to deal with three main considerations: (1) which specific natural amenities to measure; (2) how the quality of natural amenities can be captured; and (3) which type of land use is being understood as ‘nature.’

Let us first consider the specific natural amenity that the indicator is aimed to measure because importantly, we seek to support a clear interpretation of the indicator’s *values*. These values indicate the quality of the services that the targeted amenity provides to urban inhabitants. Indeed, it is the mechanism of service provision and consumption (or enjoyment or usage) that ties the simple presence of nature and associated amenities to an individual’s well-being (Boyd and Banzhaf 2007). Such well-being related services that natural amenities provide can be identified as so-called Ecosystem Services (ESS) (Costanza et al. 1997; De Groot et al. 2002; MEA 2005). While classifications of ESS vary widely, see Turner et al. (2010), we adhere to one of the most widely cited ESS classifications described in Millennium Ecosystem Assessment (2005). The Millennium Ecosystem Assessment’s classification defines direct links to well-being for *regulating* (e.g. air quality), *provisioning* (e.g. food), and *cultural* ESS (e.g. enjoyment of views). Cultural ESS are provided by amenities that include aesthetic natural features and space for recreational activities (MEA 2005) which are at the heart of this paper.

In number two our main consideration is to capture the variation in the quality of cultural ESS that distinct natural amenities provide. It has long been established that the quality of cultural ESS follows from an individual’s judgment of a nature area’s physical features (Zube 1987). As these judgments are latent, the perceived quality of recreational or aesthetic services is not directly measurable from a nature area’s physical features, thus rendering purely objective indicators as minimally effective (Pendleton and Shonkwiler 2001). Therefore, in order to identify the presence of features which indicate that recreational or aesthetic services are provided, perceptual input is required. Perceptual input can consist of either subjective (expert) judgments that support calibration of an objective indicator (e.g. Van Herzele and Wiedemann 2003), or subjective judgments of the quality of a particular piece of nature that can be merged with (GIS) coordinate-based data on natural land use (Daams et al. 2016). In effect, we use subjective judgments by people who have been sampled from urban populations in order to account for the perceptual quality of the observed nature.

Our third consideration, after defining the amenity of interest and determining how to account for variation in the quality of the amenity’s services, is to define what is understood by ‘nature.’ The definition of particular land use features that qualify as ‘nature’ is a contested matter (Castree 2005). For example, ‘nature’ may include only land use features that are untouched by humans, or it may also encompass man-made ‘nature.’ Similarly, some people consider agricultural land ‘nature’, whereas others do not (Buijs et al. 2006; Buijs 2009). Given the seeming lack of consensus in the literature on how ‘nature’ can be defined, it seems useful that indicators for recreational and aesthetic service (cultural ESS) provision of amenities be based on a wide definition of ‘natural’ land uses. A wide definition prevents possible a priori exclusion of land uses that actually include cultural ESS-providing amenities but are not defined as ‘natural.’ For example, if agricultural land were to be excluded from the indicator by strict definition, it would neglect agricultural areas that provide high quality aesthetic amenity-services.

### Spatial Measurement Alternatives

**Fig. 1:**
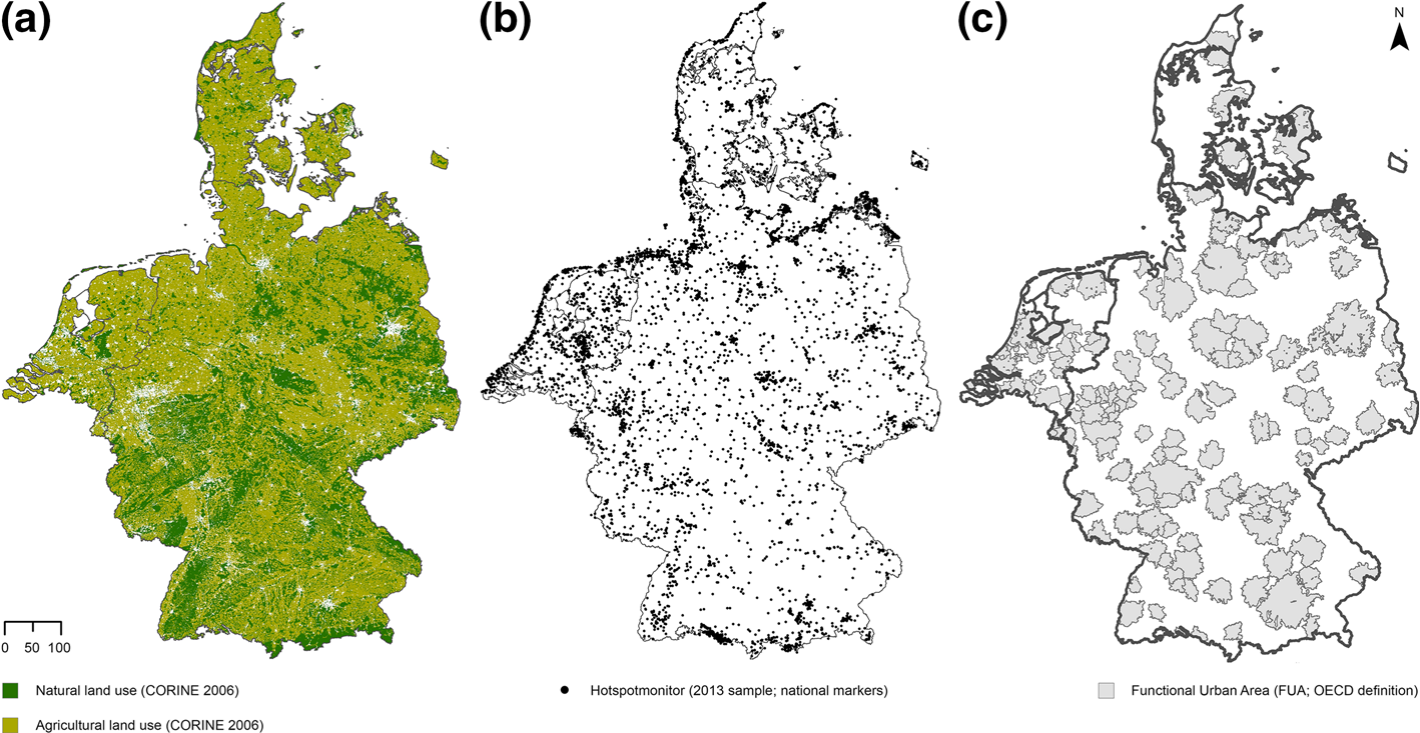
**a** ‘Natural’ and agricultural land use; **b** national Hotspotmonitor *markers* in the 2013 sample; **c** functional urban areas (FUAs)

To support our approach in this paper, we rely on data of *highly appreciated nature*, as shown in the middle of Fig. 1 (map b). These data are derived from the Hotspotmonitor survey and can be clustered (see Sect. 3.2) in order to define highly appreciated areas, that is, high-amenity nature (Sijtsma et al. 2012a, b, 2014; De Vries et al. 2013; Daams and Sijtsma 2013; Daams et al. 2016). This technique has led to a new type of land use map showing appreciated nature-related land use, but not directly to an *indicator* for urban well-being connected to these areas. To fulfill this aim requires a spatial measure that defines people’s proximity to high-amenity nature. Indeed, well-being drawn from nature may not only depend on the quality of a particular natural amenity-service, but also on the effort or resources (e.g. time, money) an individual has to invest to consume these amenity-services. The size of such efforts can be approximated with measures of the spatial relationship between consumer and observed nature. The appropriateness with which spatial relationships are captured by distinct measures depends on the type of good observed; but in the case of nature, there is no consensus on best practice (Higgs et al. 2012). Hence, as suggested by Talen and Anselin (1998), we review current practices in spatial measurement as has been observed in the literature on well-being indicators; from this basis we can choose the measurement approach that best fits our indicator’s aim.

We are most interested in the measures that are able to capture the proximity of FUA inhabitants to *high*-*amenity* nature, as this influences their enjoyment of such nature, and highlights each measure’s positive and negative aspects. In this discussion we consider six spatial measures listed in Table 1. Among these measures, two capture the presence of natural amenities at FUA-level, while the remaining four take residential locations as point of origin but can be aggregated to FUA-level. We keep in mind the three criteria outlined above that help us to select the spatial measure most appropriate for the high-amenity nature based indicator: (1) that the measure’s outcomes be easy to understand and thus support its use in spatial planning (Koenig 1980); (2) that normativity of the distance measure is minimal (Páez et al. 2012); and (3) to assess only the high-amenity natural spaces likely to be the most relevant to well-being.

**Table 1:** Key characteristics of spatial measures in the specific context of high-amenity nature

Measure	Key characteristics	Criterion		
		(1)	(2)	(3)
FUA-level	Coarse link to well-being, ignores nature outside FUA-borders			
Share of natural land use^a^	Overall ‘greenness’, comprehendible, sensitive to urban area-definition	*	*	–
Natural area per capita^a,b^	Ratio of supply and possible demand, challenging interpretation	–	*	–
Residential location based	Account for where people live as an explicit link to well-being			
Quantity within radius^c,d^	Normative, no distance-variation, limited range, comprehendible	*	–	–
Nearest distance^e,f^	Distance to likely most relevant nature, comprehendible	*	*	*
Isochronic (alternatives)	Alternatives within normative range, comprehendible, comprehensive	*	–	–
Gravity model^g^	Comprehensive, composite, challenging spatial interpretation	–	*	–

Criterion(s) indicates that a measure’s outcomes are (1) easy to understand, (2) minimally normative, and (3) assesses the high-amenity natural spaces most relevant to the well-being of a FUA’s inhabitants

* Denotes a satisfied criterion

^a^Lopes and Camanho (2013), ^b ^Zanella et al. (2015), ^c ^Maas et al. (2009), ^d ^Van den Berg et al. (2010), ^e ^Barbosa et al. (2007), ^f ^Nielsen and Hansen (2007), and ^g ^Giles-Corti and Donovan (2002)

The first of the six indicators in Table 1 captures the share of total natural land use in a FUA’s total area (Lopes and Camanho 2013). This indicator’s outcomes have a coarse link with urban inhabitants’ well-being because it does not capture whether people are likely to interact with the observed natural amenities. The potential for interaction could for example be approximated by accounting for the distance between residential locations of inhabitants and nearby high-amenity nature. Moreover, this spatial measure is particularly sensitive to how borders of urban areas are defined; this is the case because the definition of urban area borders determines whether nature at the fringe of a city is included in the indicator in a way that is (in)consistent across cities—which is a statistical concern, especially since a city’s inhabitants may benefit from nature that is located *outside* city’s borders (Guagliardo 2004).

The second FUA-level spatial measure is the amount of natural land per capita within the FUA (Lopes and Camanho 2013). This measure can capture the ratio between the supply of high-amenity nature and possible demand (the number of FUA inhabitants). The pros and cons of this measure are similar to those associated with the previously discussed measure. One relevant exception, however, is specific to the interpretation of the current indicator: its outcome values for FUAs with little amounts of high-amenity nature, and land that is mostly developed in low residential densities, may be similar to values obtained for FUAs with abundant high-amenity nature and concentrated residential areas with high population densities. Hence, the interpretation of its outcome value(s) is not straightforward with regard to green.

We now turn to the four measures that are spatially-explicit, as they are residential location-based (these measures can be aggregated to FUA-level). The first of these captures the *quantity* of natural land use within a predefined radius from people’s homes (Maas et al. 2009). A positive aspect of this measure is that it can capture the density of multiple high-amenity natural areas surrounding an individual’s home. However, as radii are pre-defined, this measure can potentially ignore high-amenity natural spaces outside the specified radius, even if the nearest space is among them. Moreover, relative differences in the distance of distinct natural areas within a given radius to a residential location are ignored. If multiple radii are specified, this would help to overcome this limitation but would necessarily lead to a complex interpretation of the measure’s outcome values.

The second measure captures the distance between a residential location and the *nearest* nature (Barbosa et al. 2007). The normativity of this measure is limited, as the distance between people’s homes and the nearest high-amenity nature is captured regardless of how wide it is, or whether it stretches beyond a FUA’s borders. But this measure ignores the presence of non-nearest high-amenity nature that may be relevant to a FUA’s inhabitants. However, this observation is of limited concern in this study, as among *high*-*amenity* natural areas, the nearest one is likely to also be the most relevant—why travel farther than the nearest excellent opportunity to enjoy attractive nature?

Our third measure is the so-called ‘isochronic’ measure, which is relevant to this discussion because of its distinctive character; furthermore, it has been proposed as a measure for quality of life in urban areas (D’Acci 2014). This type of indicator can capture the number of high-amenity natural areas accessible from a residential location within a given distance. Thus, this measure implicitly captures what is already explicitly measured in the data this study uses—the presence of high-amenity natural areas.

The fourth, and final measure is based on a *gravity* *model* (Giles-Corti and Donovan 2002). A common specification of this measure divides each natural area’s size by its distance to a single observed property, and sums the outcomes into an index value for the observed property. This measure gives a very complete indication of how close a residential location is to surrounding high-amenity nature. However, because its outcome values are a cumulative composite of sizes and distances, the spatial interpretation of this indicator is a challenging task.

We may now return to Table 1 and interpret its message: four indicators are easily understood. Four are minimally normative, but only two combine normative with being easily understandable. Thus, two serious indicator candidates remain. However, only one of these two focuses on the high-amenity natural areas which are most relevant to FUA inhabitants’ well-being: the nearest distance measure. Therefore, due to its better performance as an indicator, we will apply this measure in the remainder of the paper.

## Study Area and Data

### Natural Land Use

Our data on natural land use has been acquired from the European Environmental Agency in the form of the 2006 CORINE land cover dataset (version 17, 2013).^1^ This dataset measures land cover, including land cover by natural land use, across European countries on a 100 × 100 m grid. As such, its *overall* accuracy is assessed at 87 % (Fuller and Gaston 2009). While higher accuracy through using national data is possible, those data are not as comparable across countries as the CORINE data are. Nature is defined by any land use present in the CORINE data broadly classified as ‘natural’ (Fig. 1a). These include water bodies, wetlands, forest and semi-natural areas, agricultural areas, and artificial non-agricultural vegetated areas (e.g. parks). Such natural land cover amounts to 92, 94 and 87 % of total areas of, respectively, Germany (357,791 km^2^), Denmark (43,075 km^2^), and the Netherlands (34,976 km^2^). CORINE data for 2012, which is currently unavailable for Germany, indicates that net change in total natural land cover over 2006–2012 was −0.54 % in the Netherlands and 0.07 % in Denmark. Given the absence of a complete 2012 CORINE dataset, and the negligible benefits due to these small net changes, we deem appropriate our use of 2006 CORINE data.

### Natural Amenity-Level

High-amenity nature is operationalized using data from the Hotspotmonitor (HSM) database, which currently has international coverage for the Netherlands, Denmark and Germany (see http://hotspotmonitor.eu). The HSM is a Google Maps-based survey tool that can be described as a Participatory Geographical Information System (PPGIS) (see Brown and Kyttä 2014). The HSM asks respondents to use a so-called ‘marker’ to pinpoint a natural place on an online map that they perceive as *attractive*—for their own subjective reasons. As such, HSM data measure of Cultural Ecosystem Services (c.f. MEA 2005 in Sect. 2.1) by aesthetic and recreational amenities, essentially (MEA 2005). The HSM survey specifies to respondents that attractive natural places may be on land or water, inside or outside urban areas, and should satisfy the condition that ‘nature’ be featured in a broad sense. The HSMs measurement of perceived attractive natural places is thus based on respondents’ holistic judgment of the attributes of specific places they identify as ‘natural’ and ‘attractive’.

The designations of attractiveness (XY-point locations) observed in this study (Fig. 1b) have been gathered in a November 2013 survey among inhabitants of Dutch, Danish, and German regional capitals, whom were selected from the GfK internet-panel. The socioeconomic representation of respondents is accounted for in the sampling, by the GfK online panel, which is among the most comprehensive online panels in the observed countries. Since the survey data are used to construct a spatial measure, it is key that within the observed countries the regional distribution of respondents is balanced (De Vries et al. 2013). As a result of the stratified sampling of respondents from regional capitals, our sample includes HSM markers from respondents a from urban areas across each observed country. The (single) markers that respondents have placed pinpoint nature that is attractive specifically at the *national* scale (N = 1264 for the Netherlands; N = 1018 for Denmark; N = 4887 for Germany). An advantage of observing national scale markers is that these allow for consistent within-country comparisons of densities of markers.

The validity of Hotspotmonitor data is demonstrated in several studies, including Sijtsma et al. (2012a, b, 2014), De Vries et al. (2013), Daams and Sijtsma (2013), and Daams et al. (2016). The strength of the Hotspotmonitor data is in combining the measurement of holistic preferences with spatial precision. Indeed, capturing which specific places people perceive as *attractive nature* is challenging if one derives information only from the observable characteristics of land uses; even “expert assessments of visual landscape aesthetic quality have not proven reliable” (Daniel 2001, p. 272). Moreover, the “combination [of criteria that individuals use as they define places perceived as natural] is not fixed and may differ from space to space” (Van Herzele and Wiedemann 2003, p. 123). Both of these measurement issues are overcome by the HSM. Also, due to their standardized character, HSM data are straightforward to compare across countries.

### Urban Areas

OECD functional urban areas (FUAs) are used to delineate the borders of distinct urban areas in the Netherlands, Denmark and Germany (Fig. 1c). FUAs support robust statistical comparisons of urban areas, as they are defined based on the economic coherence of cities and their hinterland rather than on the administrative boundaries of cities (OECD 2012). The OECD database on FUAs covers 29 countries and includes more than 1100 urban areas. FUAs comprise high-density urban cores and their respective commuting zones. An urban core is identified as a cluster of high density areas, where the core is composed of all contiguous 1-square kilometer cells of at least 1500 inhabitants and having a total population of at least 50,000 inhabitants. Commuting zones are instead identified by selecting all contiguous (urban or rural) municipalities that send a minimum of 15 % of their labor force to the urban core (details are given in OECD 2012). Data used for identifying FUAs refer to the most recent available population census in each country. The way FUAs define coherent urban regions is not *entirely* new, as it compares to, for example, the Dutch COROP classification. However, the FUA definition has a unique strength, which is its potential for international implementation. Thirty-five FUAs are observed in the Netherlands, along with 109 FUAs in Germany, and 4 FUAs in Denmark.

### Population Distributions

Population distributions across FUAs in the Netherlands, Denmark, and Germany are derived from the GEOSTAT 2011 population grid (version 1.0). This grid, the result of a common project of Eurostat and the European Forum for GeoStatistics, measures total population per 1 × 1 km grid cell. These geo-referenced data include nearly 100 % of the Dutch (16.7 m), Danish (5.6 m), and German (80.2 m) populations included in the 2011 European census, and does so with high regional consistency. The spatial distribution of the measured population is represented using the ETRS89 Lambert Azimuthal Equal-Area (EPSG: 3035) coordinate system—as are all datasets used in this study. This coordinate system is compliant with the EU’s 2007 INSPIRE Directive principle that “spatial information should be comparable across member states,” and thereby supports consistent measurement of spatial indicators across our study area.

## Methodology

### Identifying High-Amenity Nature

To identify high-amenity nature, CORINE data on natural land use are combined with *clustered* Hotspotmonitor (HSM) data on the locations of attractive nature in a systematic way. This approach identifies nature perceived as attractive at national level by multiple HSM respondents, thus reflecting consensus among respondents: that the nature within each cluster is of high-amenity.

The procedure used to construct the measure of high-amenity nature is set out below. It closely follows the detailed procedure introduced in Daams et al. (2016). Broadly summarized, spatial cluster analysis is performed on HSM markers, which pinpoints natural places that people have perceived as attractive at national level. This procedure is carried out for each observed country, separately, in the following five steps: (1) A 250 × 250 m grid that covers the observed country is generated. For each separate grid cell within that grid, the density of HSM markers within a particular search radius from the grid cell is measured. The radii vary across countries, as these are scaled to account for cross-country differences in the numbers of markers observed for each square kilometer of land (see Appendix for details). For Denmark, the Netherlands, and Germany respectively, the search radii are 3.70, 2.99 and 4.87 km. Due to the scaling, the *average* density of HSM markers within a radius is consistent across countries.^2^ This ensures the cross-country consistency of the clustering process. It is noteworthy that the scaling rule uses a benchmark-search radius which is validated as optimal when classifying nature of high amenity to those who live nearby.^3^ Now that HSM marker densities are measured in the observed country, (2) a merge is performed for all contiguous grid cells for which a density of surrounding markers is observed to exceed a specific density. Specifically, the density that needs to be exceeded is the one which we would expect when markers are distributed evenly across the country’s space (a hypothetical situation where no clustering is present). (3) From the resulting clusters, only those that include multiple HSM markers are kept. Then, (4) an overlay with natural land use data is performed. This overlay removes some minimal overlap of clusters with non-natural land uses, thus making sure that the borders of clusters follow the contours of the natural land uses which they include. Finally, (5) the clusters generated separately for the Netherlands, Denmark, and Germany are merged to ensure coverage in border-regions wherever possible. This materializes the measure of high-amenity nature used here.

The numbers of places containing high-amenity nature identified in the Netherlands, Denmark, and Germany are, respectively, 77, 70 and 270. The median size of these places is 23.6 square kilometers (km^2^) in the Netherlands, 30.2 km^2^ in Denmark, and 67 km^2^ in Germany. The relatively large area in the case of Germany is consistent with nature in general being overall quite large in that country: Germany contains 329.4 km^2^ of nature, while the total areas of nature in the smaller countries of the Netherlands and Denmark are 30.5 and 40.5 km^2^, respectively. However, the sample of *high*-*amenity* nature is not restricted to large scale nature: the sizes of areas with high-amenity nature range from the 907 km^2^ of the Thüringerwald (Germany) to the 0.37 km^2^ of the Vondelpark in Amsterdam (the Netherlands).

### Indicator Specification

The indicator analyzed in this study captures for each observed functional urban area (FUA) the average Euclidean^4^ distance of its inhabitants’ residential locations to the border of the nearest high-amenity nature. Thus, the indicator accounts for the spatial distribution of people across the whole FUA territory. Residential location is taken as point of origin for the measurement of distance to the nearest high-amenity. The reason for this is that the distance from home represents “the single most important precondition” for use of nature (Van Herzele and Wiedemann 2003, p. 111). Moreover, as stated in Koenig (1980), the validity of the measurement approach used here has been established in a microeconomic empirical analysis, specifically, a study on the relation between Dutch house prices and nearby high-amenity nature (Daams et al. 2016). This is a strength of our measure, since appreciation for nature by itself offers a limited indication of well-being. Moreover, evidence from Daams et al. implies that the measure used in the present study can identify well-being from living in proximity to *high*-*amenity* nature beyond the scope of well-being indicators which are based on (objective) natural land use data alone.

Following the majority of studies that measure green space accessibility, (Higgs et al. 2012), population-weighted centroids of grid-based population data are used as a proxy for actual residential locations. While the grid-data’s 1 × 1 km resolution possibly leads to some imprecision in the measurement of distance, the level of such imprecision depends on the type of amenity to which distance is captured (Hewko et al. 2002). Given that high-amenity nature is scarce by definition, we expect that their distances relative to people’s homes in general span across several kilometers. As distance enlarges, the relative impact of within-grid-cell imprecision in distance measurement decreases, therefore, the 1 × 1 km resolution of the population data is likely sufficiently accurate for this paper’s analysis. Moreover, measurement imprecision due to the 1 × 1 km resolution is further limited because the distances between residential locations and high-amenity natural spaces are averaged out due to FUA-level aggregation.

The FUA-level aggregation of proximity outcomes for populations ensures the international comparability of the resulting indicator based on the consistent definition of ‘urban’, and also offers an additional advantage with regard to the measurement of proximity to high-amenity nature. This additional advantage stems from high-amenity nature often covering large areas of (undeveloped) land, which implies a possible negative association with population agglomerations alongside a city’s administrative borders. Our FUA-based indicator implicitly accounts for this possible association, since it measures distances to high-amenity nature for both the city-inhabitants and the inhabitants of surrounding areas that agglomerate with the city. This underpins that the indicator can be consistently compared across urban areas, also when these vary in how their populations are spatially distributed.

## Results

### Main Results

**Fig. 2:**
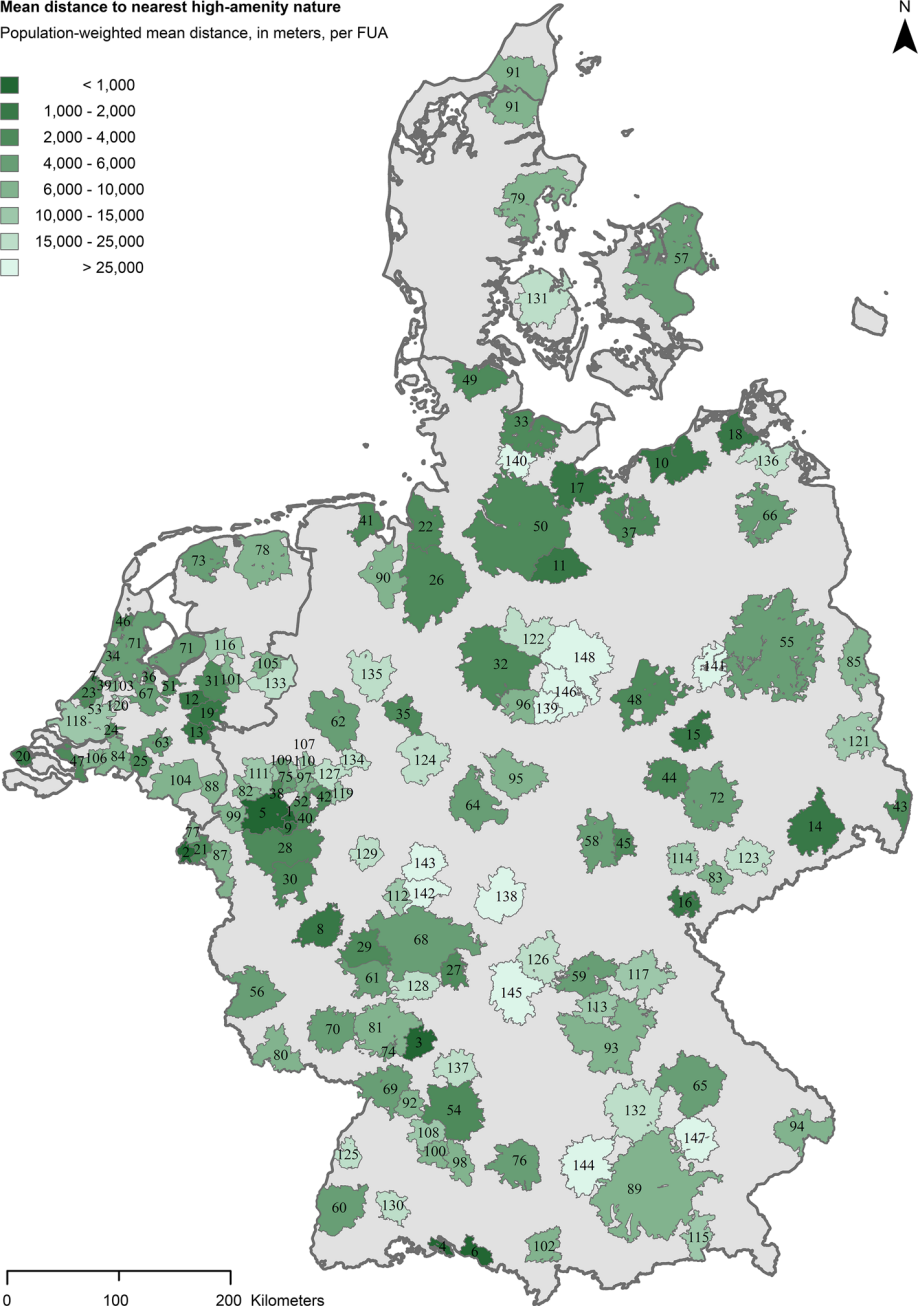
Mean population-weighted distance of each FUA population to the nearest high-amenity nature. All 148 FUAs are ranked on the basis of outcomes for this indicator (higher ranking with *lower* distance) The median is 5.8 km [(1) Solingen (2) Maastricht (3) Heidelberg (4) Konstanz (5) Düsseldorf (6) Friedrichshafen (7) Katwijk (8) Koblenz (9) Leverkusen (10) Rostock (11) Lüneburg (12) Ede (13) Nijmegen (14) Dresden (15) Dessau-Roßlau (16) Plauen (17) Lübeck (18) Stralsund (19) Arnhem (20) Middelburg (21) Heerlen (22) Bremerhaven (23) ’s-Gravenhage (24) Dordrecht (25) Tilburg (26) Bremen (27) Aschaffenburg (28) Köln (29) Wiesbaden (30) Bonn (31) Apeldoorn (32) Hannover (33) Kiel (34) Haarlem (35) Bielefeld (36) Hilversum (37) Schwerin (38) Mülheim a.d.Ruhr (39) Leiden (40) Remscheid (41) Wilhelmshaven (42) Hagen (43) Görlitz (44) Halle an der Saale (45) Weimar (46) Alkmaar (47) Bergen op Zoom (48) Magdeburg (49) Flensburg (50) Hamburg (51) Amersfoort (52) Wuppertal (53) Delft (54) Stuttgart (55) Berlin (56) Trier (57) København (58) Erfurt (59) Bamberg (60) Freiburg im Breisgau (61) Mainz (62) Münster (63) ‘s-Hertogenbosch (64) Kassel (65) Regensburg (66) Neubrandenburg (67) Utrecht (68) Frankfurt am Main (69) Karlsruhe (70) Kaiserslautern (71) Amsterdam (72) Leipzig (73) Leeuwarden (74) Speyer (75) Essen (76) Ulm (77) Sittard-Geleen (78) Groningen (79) Århus (80) Saarbrücken (81) Mannheim (82) Krefeld (83) Zwickau (84) Breda (85) Frankfurt (Oder) (86) Witten (87) Aachen (88) Venlo (89) München (90) Oldenburg (Oldenburg) (91) Aalborg (92) Pforzheim (93) Nürnberg (94) Passau (95) Göttingen (96) Hildesheim (97) Bochum (98) Reutlingen (99) Mönchengladbach (100) Tübingen (101) Deventer (102) Kempten (Allgäu) (103) Alphen aan den Rijn (104) Eindhoven (105) Almelo (106) Roosendaal (107) Recklinghausen (108) Sindelfingen (109) Oberhausen (110) Gelsenkirchen (111) Duisburg (112) Wetzlar (113) Erlangen (114) Gera (115) Rosenheim (116) Zwolle (117) Bayreuth (118) Rotterdam (119) Iserlohn (120) Gouda (121) Cottbus (122) Celle (123) Chemnitz (124) Paderborn (125) Offenburg (126) Schweinfurt (127) Dortmund (128) Darmstadt (129) Siegen (130) Villingen-Schwenningen (131) Odense (132) Ingolstadt (133) Enschede (134) Hamm (135) Osnabrück (136) Greifswald (137) Heilbronn (138) Fulda (139) Salzgitter (140) Eumünster (141) Brandenburg an der Havel (142) Gießen (143) Marburg (144) Augsburg (145) Würzburg (146) Braunschweig (147) Landshut (148) Wolfsburg]

The results show that the proximity of urban inhabitants to high-amenity nature varies widely across the observed 148 functional urban areas (FUAs) (Fig. 2; see also Table 4 in Appendix for a full ranking of all FUAs). Figure 2 shows the *population*-*weighted mean distance* (henceforth also referred to as ‘distances’) to high-amenity nature observed for each FUA, as well as the FUA’s ranking based on these distances. Lower distances to high-amenity nature are associated with higher ranks, and vice versa. The FUAs with the highest rank (distance = 0.4 km) and the lowest rank (distance = 44.9 km) to high-amenity nature are both located in Germany. Below, general results at FUA-level are scrutinized first; thereafter, a brief analysis of intra-FUA variation in distances is provided, followed by an analysis of how distances vary with urban density.

Table 2 presents the 10 FUAs for whose populations the highest and lowest distances to high-amenity nature are found. Among the ten lowest distances, distances vary between 0.4 km for Solingen (DE) as well as Maastricht (NL) to 1.4 km for Rostock (DE). The ten highest distances vary between 25.7 km for Salzgitter (DE) and 44.9 km for Wolfsburg (DE). One may notice that the four Danish FUAs are absent from Table 2—their scores are mid-range. An explanation for this is, as may be derived from Fig. 1, is that in Danish FUAs the observed high-amenity nature is often located at the edges of FUAs borders, rather than within the FUAs.^5^ As a result, there is relatively large variation in how close Danish FUA inhabitants are to high-amenity nature, but also low average distance to high-amenity nature within the FUA. This border-issue highlights the value of using nearest distance measures, as they can reach beyond FUA-borders.

**Table 2:** FUAs with the lowest and highest population-weighted mean distances to high-amenity nature

Lowest distances			Highest distances		
Rank	FUA (country)	Distance	Rank	FUA	Distance
1	Solingen (DE)	0.4	139	Salzgitter (DE)	25.7
2	Maastricht (NL)	0.4	140	Neumünster (DE)	25.8
3	Heidelberg (DE)	0.6	141	Brandenburg an der Havel (DE)	27.0
4	Konstanz (DE)	0.6	142	Gießen (DE)	28.0
5	Düsseldorf (DE)	0.8	143	Marburg (DE)	33.5
6	Friedrichshafen (DE)	0.8	144	Augsburg (DE)	34.7
7	Katwijk (NL)	0.8	145	Würzburg (DE)	35.2
8	Koblenz (DE)	1.2	146	Braunschweig (DE)	37.3
9	Leverkusen (DE)	1.3	147	Landshut (DE)	38.2
10	Rostock (DE)	1.4	148	Wolfsburg (DE)	44.9

Distances are in kilometers

When FUAs are ranked per country, the data show that in the Netherlands the three highest ranking FUAs are Maastricht (0.4 km), Katwijk (0.8 km), and Ede (1.6 km); the three lowest ranking Dutch FUAs are Rotterdam (12.6 km), Gouda (14.3 km), and Enschede (20.6 km). The highest ranking FUAs in Germany are Solingen (0.4 km), Heidelberg (0.6 km) and Konstanz (0.6 km), and the lowest ranking German FUAs are Braunschweig (37.3 km), Landshut (38.2 km), and Wolfsburg (44.9 km). The ranking of all four Danish FUAs, from high to low, is: København (4.4 km), Århus (6.5 km), Aalborg (8.0 km), and Odense (19.7 km).

Table 3 shows top and bottom ranking FUAs according to their size. Such size-based distinction can be relevant, as evidence from a study of Dutch house prices by Daams et al. (2016) suggests that, on average, inhabitants of small- and medium-sized FUAs (such as Maastricht or Heidelberg) have a slightly stronger preference for living nearby high-amenity nature than do inhabitants of large FUAs with a population above 500,000 (such as Rotterdam or Hamburg)—referred to as metropolitan FUAs hereafter.

**Table 3:** Top and low ranking FUAs, per urban class

Lowest distances^a^		Highest distances	
Rank	FUA (country)	Rank	FUA (country)
Small urban areas			
1	Solingen (DE)	53	Brandenburg an der Havel (DE)
2	Maastricht (NL)	54	Marburg (DE)
3	Konstanz (DE)	55	Landshut (DE)
Medium-sized urban areas			
1	Heidelberg (DE)	61	Würzburg (DE)
2	Koblenz (DE)	62	Braunschweig (DE)
3	Leverkusen (DE)	63	Wolfsburg (DE)
Metropolitan areas			
1	Düsseldorf (DE)	20	Rotterdam (NL)
2	Dresden (DE)	21	Dortmund (DE)
3	The Hague (NL)	22	Augsburg (DE)
Large metropolitan areas			
1	Köln (DE)	6	Frankfurt am Main (DE)
2	Hamburg (DE)	7	Amsterdam (NL)
3	Stuttgart (DE)	8	München (DE)

The FUA definition allows ‘like with like’ comparisons between ‘large metropolitan areas’ (>1,500,000 inhabitants), ‘metropolitan areas’ (500,000–1,500,000 inhabitants), ‘medium-sized urban areas’ (200,000–500,000 inhabitants), and ‘small urban areas’ (<200,000 inhabitants)

^a^Population-weighted mean distance to the nearest high-amenity nature

**Fig. 3:**
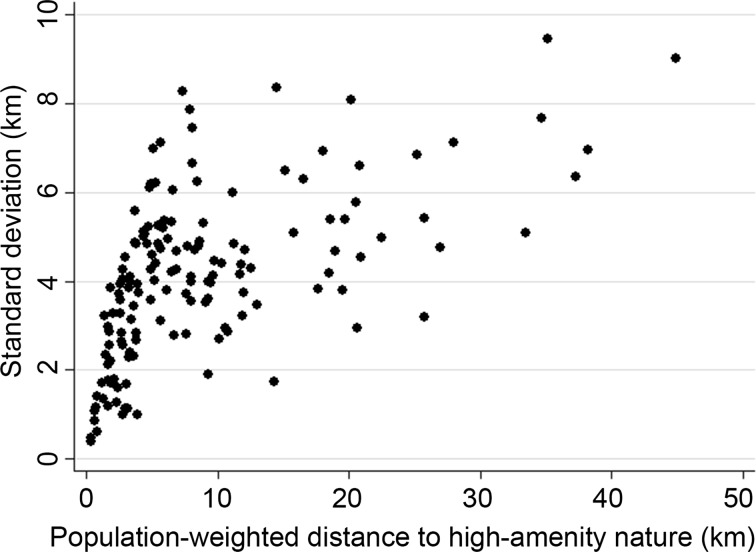
The standard deviation of FUA inhabitants’ population-weighted distances to high-amenity nature plotted against FUA-level mean distances

The proposed population-weighted indicator draws on within-FUA variation in inhabitants’ distances to high-amenity nature. Such variation can be visualized in Fig. 3, which plots the FUA-level mean of these distances against the associated standard deviation. Interestingly, in this figure’s lower left corner, a cluster of 21 FUAs can be discerned. In these FUAs inhabitants’ distances to high-amenity nature are relatively low on average (<5.0 km), and also have a relatively low standard deviation (<2.0 km). We suggest that this roughly indicates that, in these FUAs, inhabitants are close to high-amenity nature overall. A closer look at the data reveals that, of these 21 FUAs, all are classified as small- or medium-sized, except two ‘metropolitan’ FUAs (The Hague and Düsseldorf). Furthermore, of all 21 FUAs, 8 are German, and 13 are Dutch. A possible explanation for the high representation of Dutch FUAs among this subsample of FUAs may be found in population density. Consider that the average population density of Dutch FUAs within this study’s sample is 1019/km^2^, whereas the average population densities in Danish and German FUAs are 245 and 523/km^2^, respectively. The implication here is that the compactness of Dutch FUAs allows high shares of their populations to live relatively close to high-amenity nature. Given this result, we further explore the relationship between distance to high-amenity nature and population density below.

**Fig. 4:**
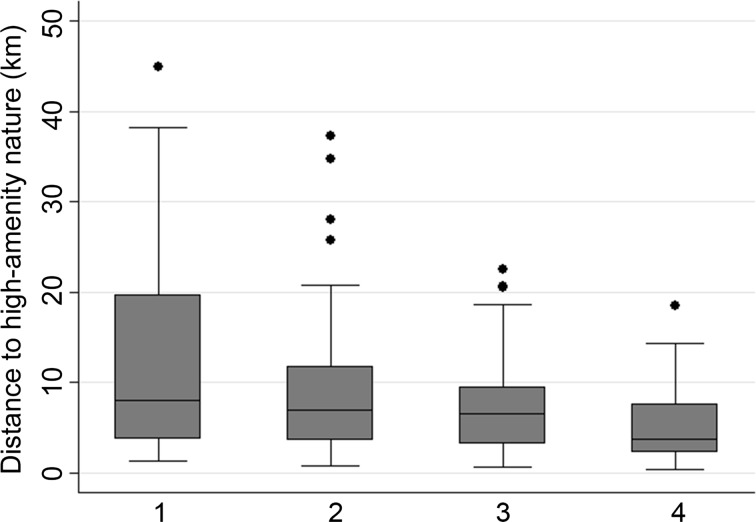
The distribution of FUAs’ (population-weighted) distances to high-amenity nature in boxplots, per population density quartile (1 = *lowest* population density; 4 = *highest* population density)

**Fig. 5:**
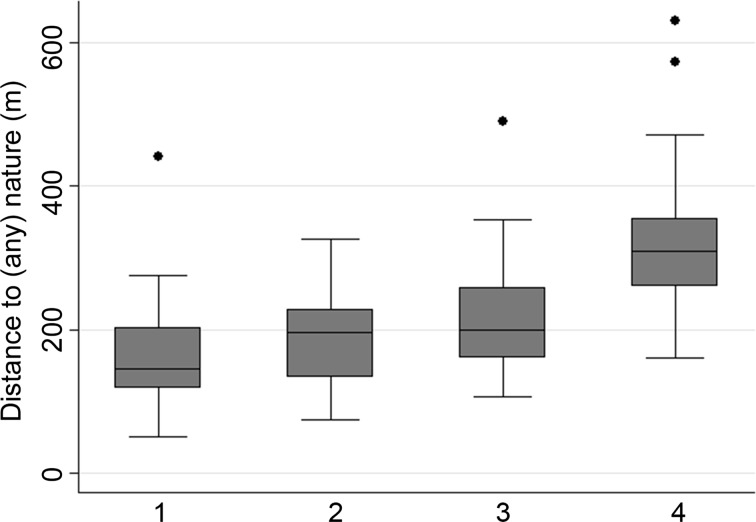
The distribution of FUAs’ (population-weighted) distances to (any) nature in boxplots, per population density quartile (1 = *lowest* population density; 4 = *highest* population density)

We consider the relationship between distance to (high-amenity) nature and population density in Figs. 4 and 5. Both figures depict the distribution of the aforementioned indicator’s outcomes for FUAs in four boxplots grouped by population density quartile (the breakpoints are densities of 226, 393, and 752 inhabitants/km^2^). Figure 4 shows per density quartile the distribution of the included FUAs’ population-weighted mean distance to the nearest high-amenity nature. FUAs in the lowest density quartile show the highest variation in distance to high-amenity nature. Moreover, among lower density FUAs are most of the FUAs for which relatively high distances are observed. Also, the inhabitants of higher density FUAs are on average closer to high-amenity nature than inhabitants of lower density FUAs. This pattern is, however, inverted when nature *in general* is observed (Fig. 5). In FUAs with a high population density, distances to the nearest nature (of any amenity-level) are on average higher than in FUAs with a low population density. This is important, since it shows that when our indicator includes subjective judgments of nature, it reveals a completely different relationship between people’s proximity to nature and population density than it does when subjective judgments are ignored.^6^ To illustrate this, the populations of FUAs in the highest density quartile, including for example’s-Gravenhage (The Hague; NL), Düsseldorf (DE), and København (DK), appear to be far from nature *in general* (given ranks on the ‘nature in general indicator’ of 142, 113, and 132, respectively; see Table S1 in the Supplementary Materials), whereas these FUAs rank quite high on the high-amenity nature indicator (i.e. ranks of 23, 5, and 57 respectively). On the other hand, the populations of some FUAs within the lower density quartile, including Wolfsburg (DE) and Würzburg (DE), are comparatively close to nature in general (given ranks of 20 and 5, respectively, on the ‘nature in general indicator’), but relative far from high-amenity nature (as they rank as 148th and 145th, respectively, on the high-amenity nature indicator).

### Sensitivity Analyses

We next perform two sensitivity analyses on the population-weighted distance to high-amenity natural spaces at FUA-level. In so doing, we restrict our FUA sample to the 35 FUAs located in the Netherlands, since only for this country are the data available that both sensitivity analyses require.

**Fig. 6:**
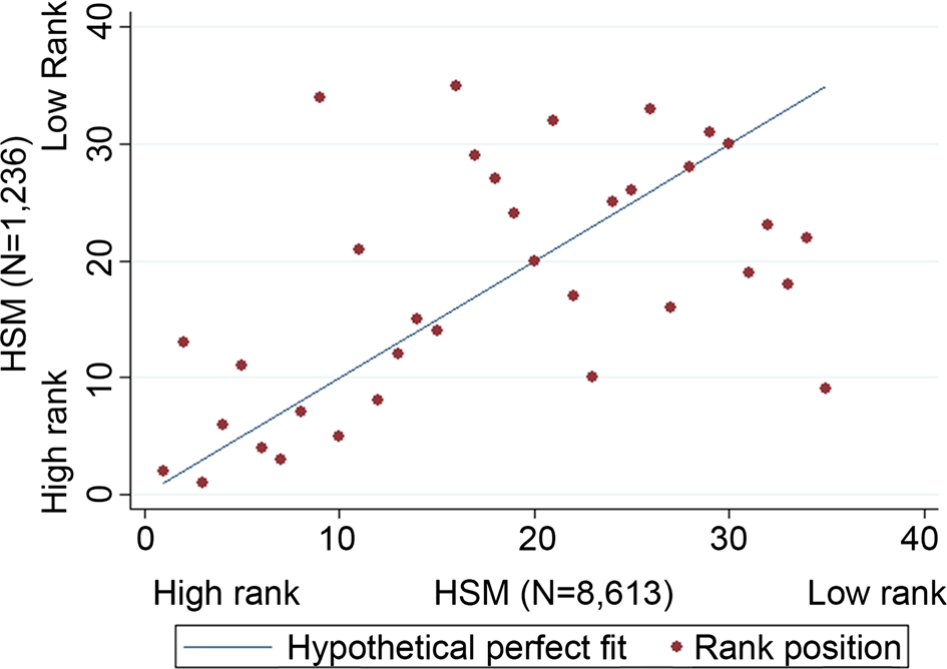
A scatterplot of two sets of rankings for FUAs: one based on the rank for population-weighted distance to high-amenity nature from an HSM sample with N = 1236 (*y* axis), the other ranking based on the similar measure but from an HSM sample with N = 8613 (*x* axis). The ‘hypothetical equal ranking’ *line* indicates a situation where FUAs would rank equally in both rankings

First, we examine how sensitive the main results for Dutch FUAs are to an increase in Hotspotmonitor (HSM) sample size—as this may identify more places with high-amenity nature than the HSM sample in the main analysis does. In this examination we establish two distinct rankings of FUAs based on the proximity of their populations to high-amenity nature. For one ranking, high-amenity nature is measured using the HSM sample (N = 1264) applied in our main analysis; for the benchmark ranking we use a sample of 8613 HSM markers that pinpoint high-amenity nature. The latter sample is based on data from all HSM surveys for the Netherlands as of August 2015. The resulting two rankings of FUAs, one for each HSM sample, are plotted against each other in Fig. 6, which also includes a ‘hypothetical equal ranking’ line. A lower vertical distance between this line and a point, which denotes a FUA’s rank, indicates a lower sensitivity of a FUA’s rank to an increase in HSM sample size.

Now let us examine the spread of points around the line in the mid and upper right part of Fig. 6. The spread indicates that the FUAs whose ranks are most sensitive to the increase in HSM sample-size rank relatively low in the main analysis. This is not surprising, since these FUAs are located in areas where high-amenity nature is scarce. Thus, when in such areas a new high-amenity natural area is identified due to an increase in HSM sample-size, then the impact on a nearby FUA’s indicator-score, i.e. its ranking, can be substantial. Consider the characteristic of the 250 *additional* places containing high-amenity nature identified using the larger HSM sample, but which remain hidden when the smaller HSM sample is used. On average, these ‘new’ places containing high-amenity nature cover 338 hectares each, whereas in the main analysis the average cover of places with high-amenity nature is 5165 hectares. In other words, observing more markers leads to observing additional, smaller areas that contain high-amenity nature in addition to those areas already identified.

The implication here is that the data in the main analysis might not reflect all high-amenity natural areas that can possibly be identified when larger HSM samples are used. Even though not all areas may be accounted for, what is most important is that the HSM sample in the main analysis identifies the natural areas that are *most* attractive, rather than natural areas of lesser attractiveness that may matter relatively less to FUA inhabitants. And although new areas will pop-up with more respondents until saturation is achieved (see Daams et al. 2016), the highest valued areas will appear first, and will thus always have a higher intensity of appreciation. The most important thing to remember about this sensitivity check is that ratios between HSM sample size and total land area associated with each country should not deviate much between countries to ensure that the high-amenity nature indicator is comparable across countries. For the outcomes of the main analysis, however, this is of limited concern; HSM samples of similar stratification are used, and small differences in how the HSM samples correspond with total land areas are already accounted for (see Appendix).

The second sensitivity analysis evaluates the appropriateness of the 1 × 1 km resolution of the Eurostat population grid used in the main analysis. In doing so, we apply a 0.1 × 0.1 km population grid covering the entire land mass of the Netherlands. This grid, which is acquired from Statistics Netherlands, measures the distribution of the Dutch population in 2011, as do the Eurostat data. Based on each of both population grids, we calculate a separate measure of FUAs’ population-weighted distance to high-amenity nature. When comparing the results, the distance measure based on 0.1 × 0.1 km grid-data is taken as a benchmark for the distances generated using the 1 × 1 km grid-data. This assessment shows that in our main analysis the measurement error in FUAs’ population weighted distance to high-amenity nature is 0.3 % on average, with a standard deviation of 2 %. Furthermore, the maximum underestimation among the calculated population-weighted is −6.4 %; this is 64 meters per km in the indicator-score. Similarly, the maximum overestimation is 4.3 %, or 43 meters per km. These minimal measurement errors clearly show that using 1 × 1 km grid data in our main analysis is not problematic.

## Discussion and Concluding Remarks

This study has introduced a well-being indicator to assess the proximity of inhabitants of 148 functional urban areas (FUAs) in the Netherlands, Denmark, and Germany to high-amenity nature. High-amenity nature has been operationalized using new systematic data on people’s perceptions of which are the locations of attractive natural areas. By doing so, the measurement approach in this paper departs from the usual assumption of a constant well-being from nature in order to approximate the ‘actual’ subjective quality of the places where people live. Evidence in Daams et al. (2016) shows that high-amenity nature may play a significant role in people’s residential location choices, and through logical association, how high-amenity nature may impact on well-being. The value added of the high-amenity nature-based indicator used in this work is that it, unlike existing studies of nature based indicators, combines the strengths of both objective and subjective measures; these strengths are, respectively, explicitness about the physical natural environment to which the indicator refers, and perceptual precision. Another advantage of the high-amenity nature based measurement approach is that it is data-efficient: it requires relatively modest samples of survey data in order to highlight the subjective quality of nature nearby distinct urban areas in multiple countries.

Results indicate that the average ‘distance’ of populations to high-amenity nature varies widely across the observed FUAs. Rankings of FUAs based on distances are provided per country and per population size-class to which they belong. Consistent comparisons of urban areas of a similar population size are possible because urban areas are defined by FUAs rather than municipal borders of cities.

A main result is that in FUAs that appear to be ‘greener’, as their populations live comparatively close to (any) natural land use, people do not necessarily live closer to *high*-*amenity* nature than people who live in FUAs which are less green. This is underlined by a low level of association between a conventional indicator, based solely on natural land use data, and our high-amenity nature indicator, which accounts for whether nature is appreciated for its attractiveness. Thus, the results that follow from this paper’s integrated objective-subjective approach to indicator measurement imply that the potential well-being of inhabitants from living near nature may be misunderstood through the use of conventional indicators alone, since well-being from living near nature may be strongly linked with that particular nature’s *attractiveness* (c.f. Daams et al. 2016). Therefore, the attractiveness-based high-amenity nature indicator introduced in this paper can be a useful complement to existing well-being indicators.

Another important finding in this study concerns the relation between the proximity of people to high-amenity nature and urban density. In FUAs with high population densities, populations are, on average, living closer to high-amenity nature than are the populations of lower density FUAs. This result is in line with the notion that in general inhabitants of lower density urban areas have to travel farther to reach certain amenities (Ewing 1997). That this holds for (high-amenity) nature is logical, since nature is the counterpart of developed land—which is extensive in low-density urban areas. Moreover, in the built structure of low-density urban areas one would expect a limited penetration by high-amenity natural amenities since these are often quite large. However, if we look at the nearness of populations of FUAs of different densities to *any* nature, the pattern is an inverse of the pattern found for high-amenity nature. Thus, higher distances to nature of *any* amenity-level are found in FUAs of higher densities. This result is not surprising: Fuller and Gaston (2009, p. 354) document “a dramatic drop in per capita green space provision in urban areas with greater population densities.” Such scarcity of nature implies a wider distance between people’s residential locations and nature. What is important here is that the results for ‘high-amenity nature’ imply that on average living in high density urban areas comes not solely at the *expense* of living as close to nature as one could in a low density urban area—as recent results from studies which define nature ‘objectively’ imply (e.g. Boyko et al. 2012; Lopes and Camanho 2013; Zanella et al. 2015). Rather, high-density living seems to enable relative high shares of FUA populations to live nearby high-amenity nature. This is an interesting finding in the long-running discussion of the ‘marriage of town and country’, invoked by Howard and carrying on to the present day (Matsuoka and Kaplan 2008): the development of high-density residential areas may allow a stronger impact of nature on people’s well-being than existing nature based indicators lead us to believe, due to the potential well-being from high-amenity nature. This new insight may add to the policy discussion of the merits and demerits of centralized and decentralized urban forms, see Breheny (1996), and how these urban forms coincide in space with the surrounding ‘country’, as represented by the often large-scale areas that comprise high-amenity nature. In addition, in the vein of findings in Carlino and Saiz (2008), our results imply that FUAs close to high-amenity nature possibly have an advantage over FUAs that are far from high-amenity nature when it comes to attracting populations.

In addition to the policy relevance of the abovementioned results with regard to urban density, the high-amenity nature based indicator can support urban planning and decision making on public investment in nature in an additional way (European Commission 2014). Information on how close, or far, FUA populations are to high-amenity nature can be used in national scale debates on regional (in)equity in the supply of high-amenity nature for recreation. A low indicator-rank may foster policy debate about increasing the supply of attractive nature near the populations of high-density FUAs, while in the case of low-density FUAs policy makers could evaluate the possible well-being benefits from local residential compaction near appreciated natural places. However, it should be clear that this study does not consider high-amenity nature as a substitute for local urban nature which may have a lower level of amenity, such as small neighborhood parks or tree cover alongside streets. Nevertheless, the data underlying the indicator can show if it is possible to distinguish among nature areas (which may be relatively large) which are more appreciated by FUA inhabitants than other nature areas. Such information can potentially increase consideration of people’s aesthetic or recreational preferences, which may indeed be tethered to their well-being, in policy decisions on nature management (Chiesura 2004). Although the potential of the indicator is apparent from the abovementioned results and their policy relevance, the indicator can be further refined.

In its current form the indicator accounts for cross-border interactions between people and natural amenities only in regions where the borders of the Netherlands, Germany, and Denmark align. Better coverage in border regions may be achieved in the future if Hotspotmonitor (HSM) data become available for more countries. Also, given Germany’s fairly large size, HSM respondents in that country can choose from a wider variety of natural areas than Dutch or Danish respondents are able to do when they designate attractive nature. As a result of this, in Germany, designations of attractiveness may be generally concentrated in the most attractive natural areas—hence, for FUAs not close to those areas, the distances of their populations to high-amenity nature is possibly overstated. To deal with this possibility, the authors have used an HSM sample which is larger for Germany than for the smaller countries Netherlands and Denmark. By doing so, country-specific idiosyncrasies inherent to any internationally comparative indicator, are mitigated, but this issue may need further attention in future studies.

As a parting thought, we would like to repeat that this paper is intended as a building block in the growing literature linking people’s well-being with natural amenities (e.g. Badland et al. 2014; Lopes and Camanho 2013; Zanella et al. 2015). We hope that, given its potentially wide applicability, the proposed indicator will enhance studies focusing on the role of nature in urban resilience, competitiveness and sustainability (Carlino and Saiz 2008; Florida et al. 2011; Glaeser et al. 2001; James et al. 2009), equitability in access to amenities (e.g. Barbosa et al. 2007), and spatial distributions of environmental quality (Brown and Kyttä 2014).

### Electronic supplementary material

Below is the link to the electronic supplementary material.

Supplementary material 1 (DOCX 77 kb)

## Appendix

As described in Sect. 4.1 in identifying high-amenity nature, specific ‘search radii’ are used; these radii are scaled per country. This scaling accounts for cross-country differences in the numbers of markers that are observed per square kilometer of land. To do so, a benchmark radius also used in a study of residential property prices in the Netherlands by Daams et al. (2016) is applied. They find that, in the context of the effect of nature on nearby residential property prices, using a radius of 1.25 km is optimal. Of course one may note that within that particular context, the optimality of the 1.25 km radius is an indirect result of the ratio between the number of observed HSM markers and the total area of the Netherlands. However, empirical evidence in Daams et al. (2016) suggests that, given their study area, the size of the sample of markers they use is quite optimal. Hence, in the current study the search radius of each country is scaled so that, on average, it contains the same number of markers as the radius in Daams et al. (2016)—assuming that markers are distributed evenly across space. Thus, the country-specific search radius *r* is identified by

1 $${\text{r}} = \sqrt {\frac{\text{H}}{{{\text{D}}\uppi}}}$$

where *H* captures the threshold number of within-radius HSM markers that leads to a grid cell being classified as ‘high-amenity natural space’ in Daams et al. (2016); *D* is the number of markers per square kilometer of land in the observed country; and *π* denotes pi.

**Table 4:** Ranking of all 148 FUAs by their score on this paper’s indicator: population-weighted distance, in kilometers, to the nearest high-amenity nature (PW-D)

Rank	FUA	PW-D	Rank	FUA	PW-D
1	Solingen (DE)	0.4	75	Essen (DE)	5.8
2	Maastricht (NL)	0.4	76	Ulm (DE)	6.0
3	Heidelberg (DE)	0.6	77	Sittard-Geleen (NL)	6.1
4	Konstanz (DE)	0.6	78	Groningen (NL)	6.2
5	Düsseldorf (DE)	0.8	79	Århus (DK)	6.5
6	Friedrichshafen (DE)	0.8	80	Saarbrücken (DE)	6.6
7	Katwijk (NL)	0.8	81	Mannheim (DE)	6.6
8	Koblenz (DE)	1.2	82	Krefeld (DE)	6.7
9	Leverkusen (DE)	1.3	83	Zwickau (DE)	6.9
10	Rostock (DE)	1.4	84	Breda (NL)	6.9
11	Lüneburg (DE)	1.4	85	Frankfurt (Oder) (DE)	7.4
12	Ede (NL)	1.6	86	Witten (DE)	7.6
13	Nijmegen (NL)	1.6	87	Aachen (DE)	7.6
14	Dresden (DE)	1.7	88	Venlo (NL)	7.7
15	Dessau-Roßlau (DE)	1.7	89	München (DE)	7.9
16	Plauen (DE)	1.7	90	Oldenburg (Oldenburg) (DE)	8.0
17	Lübeck (DE)	1.8	91	Aalborg (DK)	8.0
18	Stralsund (DE)	1.8	92	Pforzheim (DE)	8.0
19	Arnhem (NL)	1.8	93	Nürnberg (DE)	8.1
20	Middelburg (NL)	1.9	94	Passau (DE)	8.1
21	Heerlen (NL)	2.1	95	Göttingen (DE)	8.3
22	Bremerhaven (DE)	2.1	96	Hildesheim (DE)	8.5
23	`s-Gravenhage (NL)	2.2	97	Bochum (DE)	8.5
24	Dordrecht (NL)	2.3	98	Reutlingen (DE)	8.6
25	Tilburg (NL)	2.4	99	Mönchengladbach (DE)	8.9
26	Bremen (DE)	2.5	100	Tübingen (DE)	9.1
27	Aschaffenburg (DE)	2.6	101	Deventer (NL)	9.3
28	Köln (DE)	2.6	102	Kempten (Allgäu) (DE)	9.3
29	Wiesbaden (DE)	2.6	103	Alphen aan den Rijn (NL)	9.3
30	Bonn (DE)	2.7	104	Eindhoven (NL)	9.5
31	Apeldoorn (NL)	2.7	105	Almelo (NL)	9.7
32	Hannover (DE)	2.8	106	Roosendaal (NL)	9.7
33	Kiel (DE)	2.8	107	Recklinghausen (DE)	10.1
34	Haarlem (NL)	2.8	108	Sindelfingen (DE)	10.4
35	Bielefeld (DE)	2.8	109	Oberhausen (DE)	10.6
36	Hilversum (NL)	2.9	110	Gelsenkirchen (DE)	10.8
37	Schwerin (DE)	3.0	111	Duisburg (DE)	11.2
38	Mülheim a.d.Ruhr (DE)	3.1	112	Wetzlar (DE)	11.3
39	Leiden (NL)	3.2	113	Erlangen (DE)	11.7
40	Remscheid (DE)	3.2	114	Gera (DE)	11.9
41	Wilhelmshaven (DE)	3.2	115	Rosenheim (DE)	11.9
42	Hagen (DE)	3.4	116	Zwolle (NL)	12.0
43	Görlitz (DE)	3.4	117	Bayreuth (DE)	12.1
44	Halle an der Saale (DE)	3.4	118	Rotterdam (NL)	12.6
45	Weimar (DE)	3.4	119	Iserlohn (DE)	13.0
46	Alkmaar (NL)	3.6	120	Gouda (NL)	14.3
47	Bergen op Zoom (NL)	3.7	121	Cottbus (DE)	14.5
48	Magdeburg (DE)	3.7	122	Celle (DE)	15.1
49	Flensburg (DE)	3.7	123	Chemnitz (DE)	15.8
50	Hamburg (DE)	3.8	124	Paderborn (DE)	16.5
51	Amersfoort (NL)	3.8	125	Offenburg (DE)	17.7
52	Wuppertal (DE)	3.8	126	Schweinfurt (DE)	18.1
53	Delft (NL)	3.9	127	Dortmund (DE)	18.5
54	Stuttgart (DE)	3.9	128	Darmstadt (DE)	18.6
55	Berlin (DE)	4.0	129	Siegen (DE)	18.9
56	Trier (DE)	4.3	130	Villingen-Schwenningen (DE)	19.6
57	København (DK)	4.4	131	Odense (DK)	19.7
58	Erfurt (DE)	4.5	132	Ingolstadt (DE)	20.2
59	Bamberg (DE)	4.7	133	Enschede (NL)	20.6
60	Freiburg im Breisgau (DE)	4.7	134	Hamm (DE)	20.6
61	Mainz (DE)	4.9	135	Osnabrück (DE)	20.8
62	Münster (DE)	4.9	136	Greifswald (DE)	21.0
63	‘s-Hertogenbosch (NL)	4.9	137	Heilbronn (DE)	22.5
64	Kassel (DE)	5.0	138	Fulda (DE)	25.2
65	Regensburg (DE)	5.0	139	Salzgitter (DE)	25.7
66	Neubrandenburg (DE)	5.2	140	Neumünster (DE)	25.8
67	Utrecht (NL)	5.2	141	Brandenburg an der Havel (DE)	27.0
68	Frankfurt am Main (DE)	5.3	142	Gießen (DE)	28.0
69	Karlsruhe (DE)	5.3	143	Marburg (DE)	33.5
70	Kaiserslautern (DE)	5.4	144	Augsburg (DE)	34.7
71	Amsterdam (NL)	5.5	145	Würzburg (DE)	35.2
72	Leipzig (DE)	5.7	146	Braunschweig (DE)	37.3
73	Leeuwarden (NL)	5.7	147	Landshut (DE)	38.2
74	Speyer (DE)	5.7	148	Wolfsburg (DE)	44.9

## Footnotes

1 As mentioned in the text, 2012 CORINE data are unavailable for Germany. By comparing 2006 and 2012 CORINE data for the Netherlands and Denmark, we found that in our study the benefits of using 2012 data instead of 2006 data are negligible. Hence, we use the 2006 CORINE data to ensure cross-country consistency in our analysis.

2 Only the borders of the resulting high-amenity natural areas show minor deviation across countries. We suggest that is because the length of a search radius determines the distance between an outer border and the included marker nearest to it.

3 See Daams et al. (2016) empirical study of Dutch residential property prices.

4 Road-based distance metrics are possibly more refined. However, to use these in a meaningful way requires extensive data to account for multi-modal infrastructure as well as points of access to nature areas (Hewko et al. 2002; Higgs et al. 2012). Such data are not available for the observed countries, so we use Euclidean distance as a straightforward proxy for ‘actual’ distance.

5 In fact, in about 9 % of all FUAs observed in this study, for each inhabitant the nearest high-amenity nature is located outside city borders. Nevertheless, in most observed cities the majority of the population lives nearby high-amenity nature located within their city’s borders. This is illustrated by our data showing that, in the case of about 75 % of all observed cities, for more than 80 % of the city’s population, a nearest high-amenity nature is found within city borders.

6 The Pearson correlation between the high-amenity nature distance indicator and the ‘nature in general’ distance indicator based solely on natural land cover, as measured in the CORINE data is −0.24, and thus is weak. The correlated variables are log transformed to approximate normal distributions; normality is checked using Shapiro–Wilk tests. Further assessment indicates the difference in how FUAs rank on both indicators: this difference is on average 55 rank-positions, with a standard deviation of 38 rank-positions. This underlines that FUAs may rank quite differently on a nature based proximity indicator depending on whether the amenity-level of nature is accounted for.
