# Supplementary material for: Living Near to Attractive Nature? A Well-Being Indicator for Ranking Dutch, Danish, and German Functional Urban Areas
Source: Soc Indic Res. 2016 Jun 3;133(2):501–26. doi: 10.1007/s11205-016-1375-5 (PMC5569163; doi:10.1007/s11205-016-1375-5)
Supplement: Supplementary file 1 — Supplementary material 1 (DOCX 77 kb) [file 11205_2016_1375_MOESM1_ESM.docx]

**Table S1**. Ranking of all 148 FUAs based on their population-weighted distance to ‘high-amenity nature’ and ‘nature in general’, as well as our classification of each FUA by population density quartile.

| Functional Urban Area  (FUA) | High-amenity nature indicator rank | | Nature in general indicator rank | Population density quartiles  (1=low density; 4=high density) |
| --- | --- | --- | --- | --- |
| Solingen (DE) | 1 | | 116 | 4 |
| Maastricht (NL) | 2 | | 102 | 4 |
| Heidelberg (DE) | 3 | | 42 | 3 |
| Konstanz (DE) | 4 | | 87 | 4 |
| Düsseldorf (DE) | 5 | | 113 | 4 |
| Friedrichshafen (DE) | 6 | | 37 | 2 |
| Katwijk (NL) | 7 | | 122 | 4 |
| Koblenz (DE) | 8 | | 9 | 2 |
| Leverkusen (DE) | 9 | | 49 | 4 |
| Rostock (DE) | 10 | | 77 | 1 |
| Lüneburg (DE) | 11 | | 19 | 1 |
| Ede (NL) | 12 | | 120 | 2 |
| Nijmegen (NL) | 13 | | 123 | 4 |
| Dresden (DE) | 14 | | 118 | 3 |
| Dessau-Roßlau (DE) | 15 | | 74 | 1 |
| Plauen (DE) | 16 | | 86 | 1 |
| Lübeck (DE) | 17 | | 52 | 2 |
| Stralsund (DE) | 18 | | 17 | 1 |
| Arnhem (NL) | 19 | | 83 | 3 |
| Middelburg (NL) | 20 | | 28 | 3 |
| Heerlen (NL) | 21 | | 94 | 4 |
| Bremerhaven (DE) | 22 | | 88 | 1 |
| ’s-Gravenhage (NL) | 23 | | 142 | 4 |
| Dordrecht (NL) | 24 | | 139 | 4 |
| Tilburg (NL) | 25 | | 145 | 4 |
| Bremen (DE) | 26 | | 85 | 2 |
| Aschaffenburg (DE) | 27 | | 8 | 3 |
| Köln (DE) | 28 | | 91 | 4 |
| Wiesbaden (DE) | 29 | | 54 | 3 |
| Bonn (DE) | 30 | | 58 | 3 |
| Apeldoorn (NL) | 31 | | 127 | 2 |
| Hannover (DE) | 32 | | 84 | 3 |
| Kiel (DE) | 33 | | 59 | 2 |
| Haarlem (NL) | 34 | | 134 | 4 |
| Bielefeld (DE) | 35 | | 92 | 3 |
| Hilversum (NL) | 36 | | 140 | 4 |
| *Table B1 continued* | | | | |
| Schwerin (DE) | 37 | | 6 | 1 |
| Mülheim a.d.Ruhr (DE) | 38 | | 144 | 4 |
| Leiden (NL) | 39 | | 147 | 4 |
| Remscheid (DE) | 40 | | 60 | 3 |
| Wilhelmshaven (DE) | 41 | | 104 | 2 |
| Hagen (DE) | 42 | | 51 | 4 |
| Görlitz (DE) | 43 | | 78 | 1 |
| Halle an der Saale (DE) | 44 | | 130 | 2 |
| Weimar (DE) | 45 | | 76 | 1 |
| Alkmaar (NL) | 46 | | 100 | 4 |
| Bergen op Zoom (NL) | 47 | | 70 | 2 |
| Magdeburg (DE) | 48 | | 108 | 1 |
| Flensburg (DE) | 49 | | 38 | 1 |
| Hamburg (DE) | 50 | | 124 | 3 |
| Amersfoort (NL) | 51 | | 106 | 4 |
| Wuppertal (DE) | 52 | | 121 | 4 |
| Delft (NL) | 53 | | 148 | 4 |
| Stuttgart (DE) | 54 | | 44 | 4 |
| Berlin (DE) | 55 | | 146 | 3 |
| Trier (DE) | 56 | | 3 | 1 |
| København (DK) | 57 | | 132 | 3 |
| Erfurt (DE) | 58 | | 112 | 2 |
| Bamberg (DE) | 59 | | 15 | 1 |
| Freiburg im Breisgau (DE) | 60 | | 21 | 2 |
| Mainz (DE) | 61 | | 43 | 3 |
| Münster (DE) | 62 | | 40 | 2 |
| ‘s-Hertogenbosch (NL) | 63 | | 48 | 3 |
| Kassel (DE) | 64 | | 66 | 2 |
| Regensburg (DE) | 65 | | 30 | 1 |
| Neubrandenburg (DE) | 66 | | 47 | 1 |
| Utrecht (NL) | 67 | | 136 | 4 |
| Frankfurt am Main (DE) | 68 | | 50 | 3 |
| Karlsruhe (DE) | 69 | | 41 | 3 |
| Kaiserslautern (DE) | 70 | | 36 | 1 |
| Amsterdam (NL) | 71 | | 131 | 4 |
| Leipzig (DE) | 72 | | 107 | 2 |
| Leeuwarden (NL) | 73 | | 96 | 2 |
| Speyer (DE) | 74 | | 71 | 3 |
| Essen (DE) | 75 | | 126 | 4 |
| Ulm (DE) | 76 | | 22 | 2 |
| Sittard-Geleen (NL) | 77 | | 65 | 4 |
| Groningen (NL) | 78 | | 117 | 2 |
| *Table B1 continued* | | | | |
| Århus (DK) | 79 | | 98 | 1 |
| Saarbrücken (DE) | 80 | | 53 | 3 |
| Mannheim (DE) | 81 | | 72 | 3 |
| Krefeld (DE) | 82 | | 138 | 4 |
| Zwickau (DE) | 83 | | 95 | 2 |
| Breda (NL) | 84 | | 135 | 3 |
| Frankfurt (Oder) (DE) | 85 | | 62 | 1 |
| Witten (DE) | 86 | | 80 | 4 |
| Aachen (DE) | 87 | | 56 | 3 |
| Venlo (NL) | 88 | | 79 | 3 |
| München (DE) | 89 | | 110 | 3 |
| Oldenburg (Oldenburg) (DE) | 90 | | 75 | 2 |
| Aalborg (DK) | 91 | | 64 | 1 |
| Pforzheim (DE) | 92 | | 25 | 3 |
| Nürnberg (DE) | 93 | | 109 | 2 |
| Passau (DE) | 94 | | 1 | 1 |
| Göttingen (DE) | 95 | | 57 | 1 |
| Hildesheim (DE) | 96 | | 29 | 2 |
| Bochum (DE) | 97 | | 137 | 4 |
| Reutlingen (DE) | 98 | | 82 | 2 |
| Mönchengladbach (DE) | 99 | | 101 | 3 |
| Tübingen (DE) | 100 | | 34 | 3 |
| Deventer (NL) | 101 | | 81 | 2 |
| Kempten (Allgäu) (DE) | 102 | | 18 | 1 |
| Alphen aan den Rijn (NL) | 103 | | 119 | 4 |
| Eindhoven (NL) | 104 | | 129 | 3 |
| Almelo (NL) | 105 | | 90 | 2 |
| Roosendaal (NL) | 106 | | 105 | 3 |
| Recklinghausen (DE) | 107 | | 103 | 4 |
| Sindelfingen (DE) | 108 | | 33 | 3 |
| Oberhausen (DE) | 109 | | 143 | 4 |
| Gelsenkirchen (DE) | 110 | | 133 | 4 |
| Duisburg (DE) | 111 | | 111 | 4 |
| Wetzlar (DE) | 112 | | 2 | 2 |
| Erlangen (DE) | 113 | | 26 | 2 |
| Gera (DE) | 114 | | 67 | 2 |
| Rosenheim (DE) | 115 | | 11 | 2 |
| Zwolle (NL) | 116 | | 89 | 3 |
| Bayreuth (DE) | 117 | | 27 | 1 |
| Rotterdam (NL) | 118 | | 115 | 4 |
| Iserlohn (DE) | 119 | | 12 | 3 |
| Gouda (NL) | 120 | | 125 | 4 |
| *Table B1 continued* | | | | |
| Cottbus (DE) | | 121 | 141 | 1 |
| Celle (DE) | | 122 | 35 | 1 |
| Chemnitz (DE) | | 123 | 97 | 3 |
| Paderborn (DE) | | 124 | 31 | 1 |
| Offenburg (DE) | | 125 | 24 | 2 |
| Schweinfurt (DE) | | 126 | 7 | 1 |
| Dortmund (DE) | | 127 | 114 | 4 |
| Darmstadt (DE) | | 128 | 68 | 3 |
| Siegen (DE) | | 129 | 14 | 2 |
| Villingen-Schwenningen (DE) | | 130 | 46 | 2 |
| Odense (DK) | | 131 | 99 | 1 |
| Ingolstadt (DE) | | 132 | 13 | 1 |
| Enschede (NL) | | 133 | 128 | 3 |
| Hamm (DE) | | 134 | 73 | 3 |
| Osnabrück (DE) | | 135 | 45 | 2 |
| Greifswald (DE) | | 136 | 55 | 1 |
| Heilbronn (DE) | | 137 | 39 | 3 |
| Fulda (DE) | | 138 | 4 | 1 |
| Salzgitter (DE) | | 139 | 32 | 2 |
| Neumünster (DE) | | 140 | 63 | 1 |
| Brandenburg an der Havel (DE) | | 141 | 69 | 1 |
| Gießen (DE) | | 142 | 23 | 2 |
| Marburg (DE) | | 143 | 10 | 1 |
| Augsburg (DE) | | 144 | 93 | 2 |
| Würzburg (DE) | | 145 | 5 | 1 |
| Braunschweig (DE) | | 146 | 61 | 2 |
| Landshut (DE) | | 147 | 16 | 1 |
| Wolfsburg (DE) | | 148 | 20 | 1 |
| *Note*: the breakpoints for the population density quartiles are densities of 226, 393, and 752 inhabitants/km². | | | | |
